# Comparison of Wireless Continuous Axillary and Core Temperature Measurement after Major Surgery

**DOI:** 10.3390/s24144469

**Published:** 2024-07-10

**Authors:** Anders Blom Nathansen, Jesper Mølgaard, Christian Sylvest Meyhoff, Eske Kvanner Aasvang

**Affiliations:** 1 Department of Anesthesiology, Center for Cancer and Organ Diseases, Rigshospitalet, University of Copenhagen, Blegdamsvej 9, 2200 Copenhagen, Denmarkeske.kvanner.aasvang.01@regionh.dk (E.K.A.); 2 Department of Anesthesia and Intensive Care, Bispebjerg Hospital, University of Copenhagen, 2400 Copenhagen, Denmark; 789456@regionh.dk; 3 Department of Clinical Medicine, University of Copenhagen, 2200 Copenhagen, Denmark

**Keywords:** vital signs, fever, temperature, wireless monitoring, axillary, validation

## Abstract

Background: Temperature is considered one of the primary vital signs for detection of complications such as infections. Continuous wireless real-time axillary temperature monitoring is technologically feasible at the general ward, but no clinical validation studies exist. Methods: This study compared axillary temperature with a urinary bladder thermometer in 40 major abdominal postoperative patients. The primary outcome was changes in axillary temperature registrations. Secondary outcomes were mean bias between the urinary bladder and the axillary temperatures. Intermittent frontal and tympanic temperature recordings were also collected. Results: Forty patients were monitored for 50 min with an average core temperature of 36.8 °C. The mean bias was −1.0 °C (LoA −1.9 to −0) after 5 min, and −0.8 °C (LoA −1.6 to −0.1) after 10 min when comparing the axillary temperature with the urinary bladder temperature. After 20 min, the mean bias was −0.6 °C (LoA −1.3–0.1). During upper arm abduction, the axilla temperature was reduced to −1.6 °C (LoA −2.9 to −0.3) within 1 min. Temporal skin temperature measurement had a resulted in a mean bias of −0.1 °C (LOA −1.1 to −1.0) compared with central temperature. Compared with the mean tympanic temperature, it was −0.1 °C (LoA −0.9 to −1.0) lower than the urinay bladder temperature. Conclusions: Axillary temperature increased with time, reaching a mean bias of 1 °C between axillary and core temperature within 5 min. Opening the axillary resulted in rapidly lower temperature recordings. These findings may aid in use and designing corrections for continuous axillary temperature monitoring.

## 1. Introduction

Temperature is one of the primary vital signs and included in standard manual screening tools such as the National Early Warning Score (NEWS), commonly used in several countries as a clinical assessment of patients [1]. Fever can be the only deviating vital sign when patients are developing septicemia, for instance, in cases of febrile neutropenia [2,3].

In the general ward, the current clinical standard is measuring patient’s vital sign values once every 8 h unless there are signs of patient deterioration [4]. This can potentially result in undetected vital sign deteriorations and complications lasting for hours before staff intervenes. Patients with postoperative infections such as surgical site infection, pneumonia, and urinary tract infections have an estimated >4.5-fold increased incidence of mortality [5]. Patients at risk of these complications could benefit from continuous vital sign monitoring (CVSM), which may detect developing fever at an earlier stage than the current clinical standard being intermittent monitoring of vital signs NEWS. CVSM is a technology with mounting evidence for improved clinical outcomes such as significantly reduced number of complications, admissions to the intensive care unit, and the need for rapid response calls [4,6,7].

However, CVSM has until recently been reserved for high-demand departments such as the intensive care unit (ICU), the post-anesthetic care unit (PACU), or the emergency department. The reasons being a combination of wired solutions keeping patients bedridden and a high alert frequency from simple threshold alerts especially problematic at the general ward with the low nurse: patient ratio and risk for alarm fatigue.

The gold standard for measuring core temperature is recordings either from a urinary bladder catheter, pulmonary artery catheter, or esophageal thermistor. However, these modalities are invasive and normally reserved for intraoperative or critically ill patients [8].

Temperature recording outside the ICU, PACU, and OR mainly consists of non-contact infrared thermometers (NCIFT) such as tympanic or temporal skin thermometers, but these are less reliable, and the recommendation is weak [8]. Tympanic infrared thermometers provide an estimate of the core temperature by measuring the radiant energy from the tympanic membrane. This can be influenced by ear wax, inflammation of the ear canal, and the operator’s technique [8]. NCIFT can also be used on the forehead by measuring the temperature of the temporal artery. This method is also associated with uncertainty, being affected by for example ambient room temperature and sweating.

Recent advances have resulted in wireless sensor technology being implemented at the general ward for CVSM. These systems are ideally supported by real-time AI analysis of the relation to complications to reduce the number of irrelevant alerts which are causing alarm fatigue [4]. Incorporating temperature assessment may allow for earlier identification of early signs of fever and allow for preventive measures to reduce complication severity; i.e., an early detection of pneumonia may reduce the risk for transition into septicemia [9]. Recently, wireless devices have been introduced that are capable of measuring axillary temperature continuously with real-time alerts. The axilla is an obvious candidate for noninvasively measuring temperature continuously, as it is an area of skin in an anatomical “pit”, potentially protecting the device from fluctuations in ambient temperature. It is also a more hygienic choice compared to a rectal or oral device. If clinical decisions regarding treatment should be based on information from these readings, accurate measurements of discrepancy from core temperature are needed. Thus, although devices are proven to be technically very accurate with a median deviation from the actual temperature being 0.1 °C under laboratory settings [10], few studies exist on their accuracy in clinical settings, including description of time to reach an acceptable deviation from temperature measurements by wireless axillary thermometers.

The current study aimed to compare the axillary temperature measured by a continuous wireless device to core body temperature measured in the urinary bladder during PACU admission after major abdominal surgery and secondarily to describe the time relation before a difference ≤ 1 degree Celsius between the two measurements occurred. The hypothesis was a systematic bias towards lower axillary temperature readings that would be reduced over time with closed axilla.

## 2. Materials and Methods

The study was approved as a clinical quality development project by the local legal datahandling authorities (Workzone no.: 23050470), and patient consent was obtained after oral information and documented in the hospital charts accessible to the patient.

### 2.1. Study Population

Adult patients (≥18 years) with a urinary bladder thermometer and an expected stay of >2 h from start of monitoring in the PACU were included after major abdominal surgery.

### 2.2. Study Set-Up

Patient temperatures were recorded whilst the patient was lying supine in bed undergoing standard procedure specific treatment at the PACU. All data were collected by a single investigator throughout the study period. Ambient room temperature was kept at 21 degrees, during the daytime. Per local standards, patients were not transferred to the PACU with temperatures < 36 °C. All patients were also given 1 g paracetamol from the start of surgery and every 6 h at the PACU.

The core temperature was measured by a urinary bladder thermometer (Mon-a-therm™ Foley with Temperature Sensor 400™, Medtronic, Minneapolis, MN, USA) transmitting continuously to a bedside screen.

The primary comparator was the Lifetemp, a wireless continuous axillary thermometer (Isansys Lifecare, Oxfordshire, UK) with minute tramsission and a proven mean bias of <−0.1 °C compared to actual temperature under laboratory settings per the device datasheet. The sensor is placed high in the axilla, and the battery and Bluetooth transmitter are located 10 cm below on the truncus. The device is adhered to the skin via a medical-grade silicone gel [10]. Secondary comparators were intermittent recordings by an ear thermometer (Covidien Genius 2—Covidien Medtronic, Minneapolis, MN, USA) which provides a result within 2 s by choosing from 100 readings and finding the most accurate one [11]. We also investigated a non-contact infrared thermometer NCIFT Microlife NC200 (Widnau, Switzerland). This thermometer works by registering infrared radiation from the skin and then provides an estimate of central temperature when the device is the correct distance from the skin for 3 s [12]. It has an integrated algorithm which incorporates readings of the ambient room temperature and corrects the measured patient temperature, which is the one reported in this study. The specific correction factor is undisclosed by the manufacturer, despite direct request.

### 2.3. Monitoring Procedure

The Lifetemp patch was applied in the axilla, and the patient was instructed to keep the arm close to the lateral side the body, thus keeping the axilla closed for 20 min. Temperatures from the urinary bladder, axilla, forehead, and ear were registered/measured at 1, 5, 10, and 20 min. Patients were then asked to extend the arm above the head or to the side, thus opening the axilla for 5 min. Temperatures were registered after 1 and 5 min with open axillae. This was repeated for a total of two series and a total of 12 measurements for each patient with all four devices.

#### 2.3.1. Statistical Analyses and Outcomes

Sample size: No similar studies existed and we aimed to include 40 patients with full data to allow for one outlier (2.5%) outside each side of the assumed 95% normal distribution.

##### The Primary Outcome Was Axillary Temperature Development

Secondary outcomes were mean bias between the urinary bladder and the axillary thermometer measurements at 1, 5, 10, and 20 min with 95% limits of agreement (LoA). Tertiary outcomes were mean bias between the urinary bladder and the forehead and ear measurements.

Data were presented with descriptive statistics. We used the Bland and Altman method for repeated measurements to compare modalities [13]. We calculated root mean square deviation providing an average of the deviation from our reference i.e., the bladder temperature.

All data analyses were performed in Microsoft Excel (Microsoft 365 MSO Version 2302 Build 16.0.16130.20942).

## 3. Results

The 40 patients who completed the study had an average age of 66 years (range 30–82), and 80% were male. Patient demographics are summarized in Table 1. A total of 480 temperature measurements were recorded for each device. Patients had a mean temperature of 36.8 °C with no cases of fever (>38.0 °C), but two cases of hypothermia (<36.0 °C) were observed, measured by our reference bladder catheter.

### 3.1. Lifetemp Compared to Bladder:

**Figure 1:**
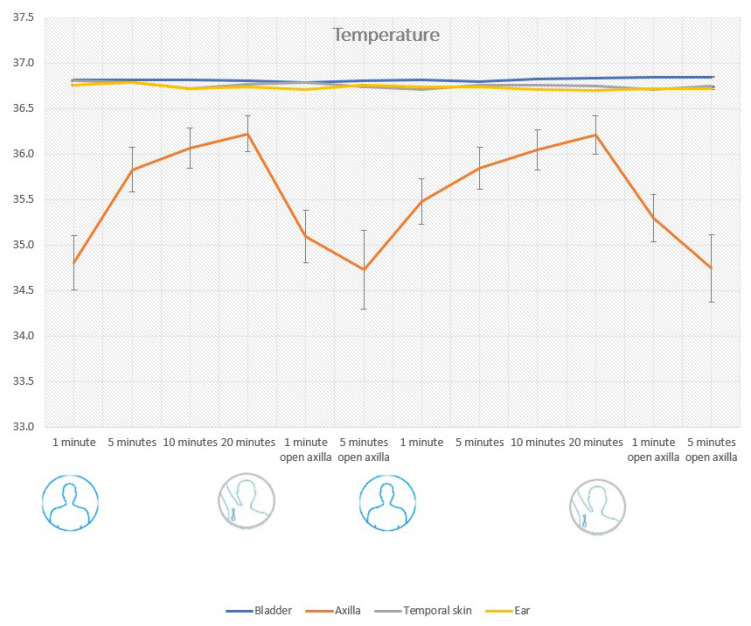
Showing how the temperature changes in Celsius in relation to how long the arm is kept close to the body and after opening the axilla. Data points are including standard deviation.

**Figure 2:**
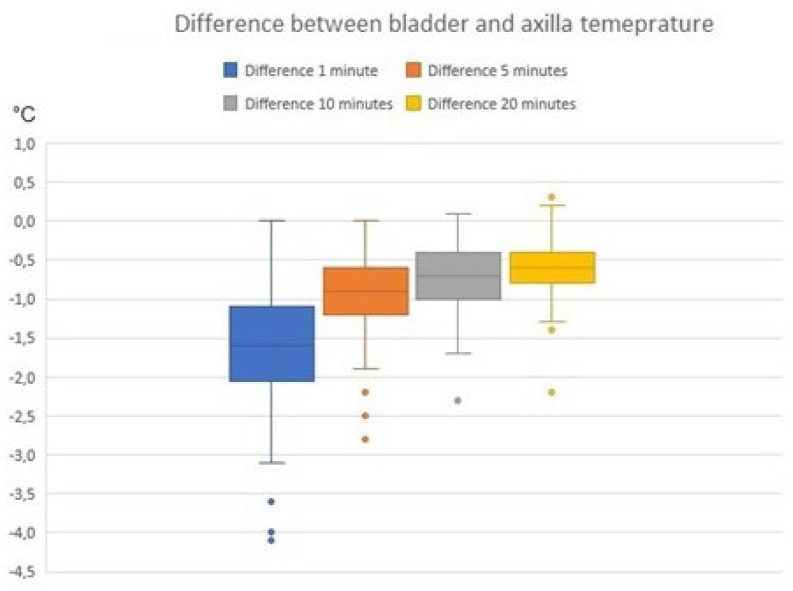
Boxplots showing the difference between axillary and bladder temperature at different time intervals with closed axillae; y axis is the temperature difference in Degrees Celsius (°C).

**Figure 3:**
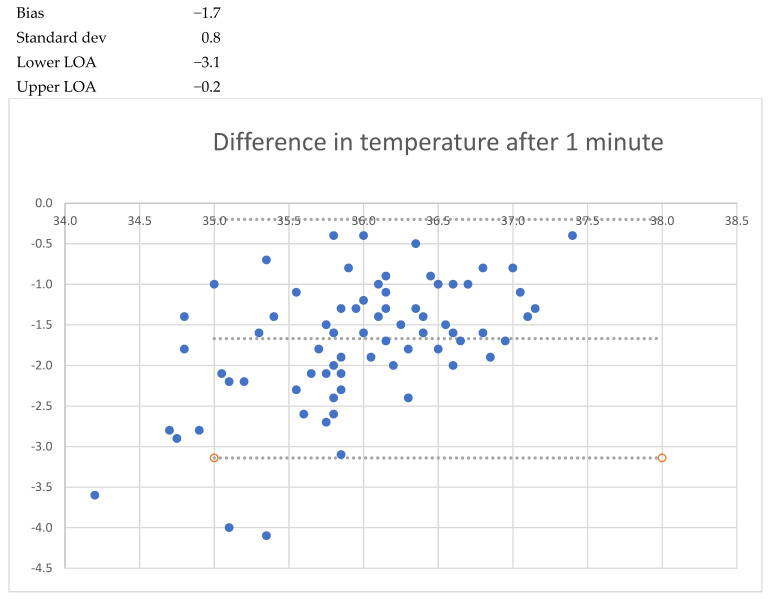
Bland and Altmann plots of agreement between measurements of axillary and urinary bladder temperatures after 1 min with closed axilla. Upper dotted line shows the upper limit of agreement, the middle dotted line the mean bias, and the lowest dotteld line the lower limits of agreement.

**Figure 4:**
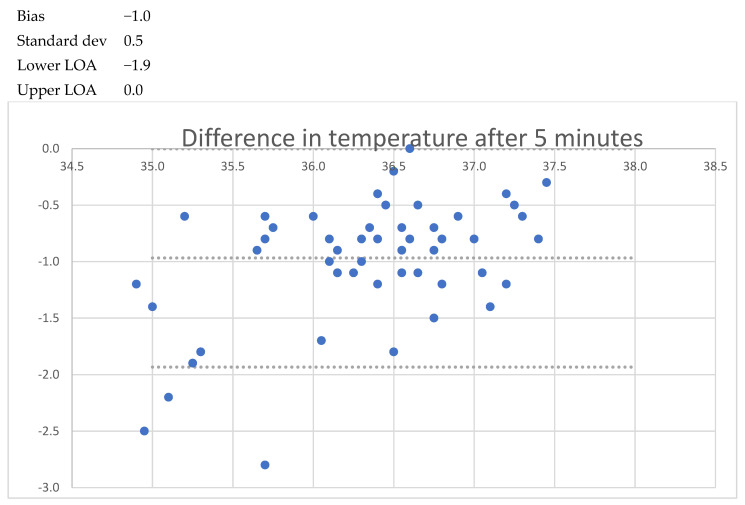
Bland and Altmann plots of agreement between measurements of axillary and urinary bladder temperatures after 5 min with closed axilla. Upper dotted line shows the upper limit of agreement, the middle dotted line the mean bias, and the lowest dotteld line the lower limits of agreement.

**Figure 5:**
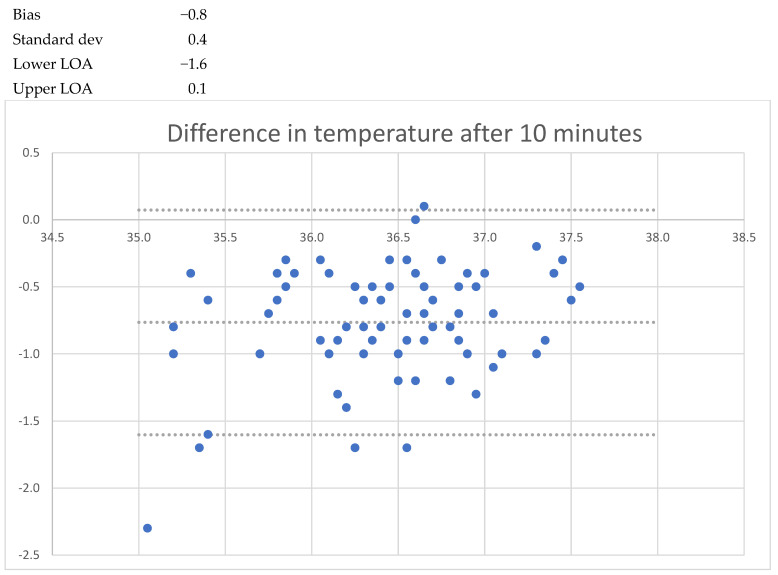
Bland and Altmann plots of agreement between measurements of axillary and urinary bladder temperatures after 10 min with closed axilla. Upper dotted line shows the upper limit of agreement, the middle dotted line the mean bias, and the lowest dotteld line the lower limits of agreement.

**Figure 6:**
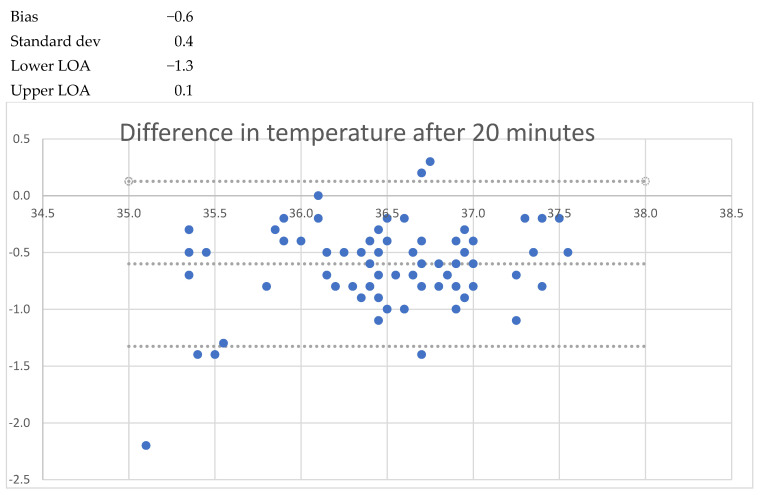
Bland and Altmann plots of agreement between measurements of axillary and urinary bladder temperatures after 20 min with closed axilla. Upper dotted line shows the upper limit of agreement, the middle dotted line the mean bias, and the lowest dotteld line the lower limits of agreement.

After one minute of recording, the mean bias was −1.7 °C (LoA −3.1 to −0.2); after five minutes, the mean bias was −1.0 °C (LoA −1.9 to 0.0); and after 20 min the mean bias was − 0.6 °C (LoA −1.3 to 0.1). When the patient was asked to lift the arm, a lower temperature was seen, decreasing to −1.6 °C (LoA 0.6 to −2.9) after 1 min. After 5 min, the mean bias was 1.9 °C (LoA −4.0 to 0.2) with a standard deviation of 1.1 °C. No cases of fever (urinary bladder temp > 38.0 °C) were registered, but two cases of hypothermia (35.5 °C and 35.7 °C) were registered. These findings are detailed in Table 2 and Figure 1, Figure 2, Figure 3, Figure 4, Figure 5 and Figure 6.

### 3.2. Tympanic Thermometer Compared to Bladder

Tympanic temperature measurements from the ear thermometer (Coviden genius 2) resulted in a mean bias of −0.1 °C (LoA −0.9 to −1.0) lower than the central temperature. Results are summarized in Table 2 and Figure 1.

### 3.3. Temporal Skin Thermometer Compared to Bladder

The temporal skin temperature measurements resulted in a mean bias of −0.1 °C (LoA −1.1 to 1.0) Results are summarized in Table 1 and Figure 1.

## 4. Discussion

The primary finding from the present study was a continued rise in axillary temperature during closed axilla, reaching a mean bias of ≤1 degrees Celsius within 5 min between continuous axillary and core body temperature (urinary bladder).axilla. Opening the axilla resulted in large deviations between axillary and core temperature within 1 min. Our findings are comparable to another study comparing a wireless continuous axillary temperature measurement to a urinary bladder thermometer in 36 febrile patients defined as temperature >38 °C, where a mean bias of −1.11 °C LoA (−0.98 to 3.19) was observed after more than 5 min [14].

The main reason for temperature monitoring is to detect fever or hypothermia due to the relation to underlying pathologies such as infections and the direct adverse effect on physiology.

Thus, fever is a common response to infection and inflammation; it is developed through several humoral and neurological mechanisms [15]. The body’s core temperature is regulated centrally in the hypothalamus with anormal setpoint around 37 °C [16].

Pathophysiologically, fever, or hypothermia from infections is the results of the immune systems exposureto exogenous pyrogens such as lipopolysaccharids from bacterial cell walls, resulting in release of endogenous pyrogens in the form of cytokines. These affect the cerebral organum vasculosum of the lamina terminalis directly because it lacks the blood–brain barrier as well as indirectly through the afferent limb of the vagus nerve. This increases the production of prostanoids, impacting the pre-optic nucleus by slowing the fire rate of heat sensitive neurons thus increasing the thermal setpoint resulting in changes in body temperature [17]. However, not all infections cause fever, and some even result in hypothermia [18]. Although fever is not a well-defined parameter, the Center for Disease Control and Prevention states that fever is a temperature of 38.0 °C or higher [7], clinicians are often faced with patients stating that they feel fever at lower temperatures, and indeed there seems to be a paucity in the area of normal temperature variations [16]. Nonetheless, the detection of fever is considered pivotal in the diagnosis of infection as it may allow for preventing patient deterioration and monitor treatment efficacy

One small study with 31 readings investigated discrepancy between axillary (electronic spot measurement) and core temperature (pulmonary artery catheter) and found a difference of 0.33 °C after being left in place for 10 min [19]. A difference between axillary and pulmonary artery temperature of − 0.27 °C (±0.45 °C) was found in 42 patients, 39 of whom where sedated and mechanically ventilated, after a wired axillary thermometer had been in place for 2–3 min [20]. These studies were conducted on ICU patients who likely had had their axilla closed for extended periods, which may account for the smaller mean bias compared to our findings due to higher axillary starting temperatures.

Not only do axillary measurements approach the core temperature over time, the limit of agreement also becomes increasingly narrower, suggesting better reliability the longer the axilla is closed. American guidelines suggest adding 1 degree Celsius when measuring temperature in the axilla, although the method is not recommended [8]; in Denmark adding 1.2 degrees °C is recommended [21].

The consequences of autocorrection may be false positive alerts of fever in cases of closed axilla.

However, the accuracy of axillary temperature is contingent upon the arm remaining close to the body. When the arm is opened for just one minute, axillary measurements register −1.9 °C (LoA −4.0 to 0.2) lower than core temperature, potentially leading to false hypothermia alerts, especially if patients are mobile. A suggestion could be implementing a filter in the warning system to trigger alarms only for gradual temperature drops over extended periods. Our data suggest that axillary temperature could inherently measure −1.3 °C (lower limit of agreement) lower than central temperature, even when the thermometer has been attached for 20 min, emphasizing the risk of false hypothermia diagnosis. As such, the axillary temperature lies between the peripheral skin temperature and the central temperature even after 20 min with a fully closed axilla, unlikely to occur throughout the full day in mobile patients.

We found that the ear thermometer Coviden genius 2 had accurate measurements compared to bladder temperature. With an RMSD of 0.4, this was the most accurate method. The NCIFT Microlife nc200 used on the skin of the temporal region had the same mean bias of −0.1 degree; however, the RMSD was 0.6, suggesting less consistency. Both thermometers are very easy to use and offer a rapid and accurate measurement. Current guidelines, relying on spot measurements, suggest adding 0.5 degrees when measuring the tympanic- or temporal skin temperature. However, there exists no exact consensus, and this may be an overcompensation potentially leading to overtreatment of fever given that a correction is integrated in the software [10,21].

Zero-heat-flux thermometry is another way of measuring temperature non-invasively, but not investigated in the current study. By heating the tissue surrounding the sensor, an isothermal tunnel is created. A comparison study found an average overall difference of −0.3 °C (LoA ± 0.88) compared with a pulmonary artery catheter. This method offers continuous measurements but is dependent on a power supply, thus keeping the patient bedridden [22].

Strengths: A major strength of the current study was the inclusion of the urinary bladder temperature as gold-standard. In addition, all measurements were made on postoperative patients in the PACU, representing a group of patients the equipment is intended for. The stringent methodology allowed us to identify the weakness of the open axilla and the related caveats for future clinical use. Temperatures for the four different modalities were registered 12 times for each patient, resulting in 480 total datapoints for each modality comparable to bladder temperature.

Limitations: The main limitation of the current study is the absence of patients with fever, not allowing us to explore the accuracy in the higher temperatures. Our study included 40 patients, which may seem like a relatively small sample size, but was adjusted for by the repeated measurements. Finally, the performance in patients at the general medical and surgical wards during activity was not investigated and it may likely result in larger LoA when patients are ambulating.

From a clinical perspective, the axillary measurement cannot serve as a direct measurement of the central temperature, even with a fully closed axilla. This results in the need for correcting the temperature by 1 degree to detect cases of potential fever, but with the inherent risk of over alerting in those cases. However, we believe it is safer to err on the safe side in this case. The study also shows that the device is not suitable for detection of hypothermia due to the risk of incorrect low measurements.

Perspectives: The current findings suggest that caution should be taken when using the Lifetemp device, especially if corrective factors are not implemented. On the other hand, the device may identify cases of fever much earlier than current clinical standards, consisting of intermittent monitoring 8–12 h apart [1]. The intermittent nature also risk failing to detect fever due to fluctuations in temperature. As such, continuous temperature recording may allow for early interventions to reduce complication severity. Adding other modalities such as pulse or respiration rate to the temperature information may in time allow for more individualized thresholds also or serve as triggers for adding temperature recording as a secondary vital sign upscaling. Based upon our results, work should also focus on combining the accuracy from tympanic membrane thermometers with wireless devices measuring, and transmitting data, continuously.

## 5. Conclusions

A one-degree Celsius mean difference between axillary and core temperature was achieved within 5 min and further reduced from there on. Opening the axilla resulted in rapid loss of temperature. These findings may aid in designing corrections for axillary recordings and continuous wireless temperature monitoring.

## Figures and Tables

**Table 1:** Basic characteristics of the patients: Study population *n* = 40.

Group		
Male	31.0	80%
Female	9.0	20%
Mean age at surgery:	66.53	
Age Range:	30–82	
Type of surgery:		
Liver resection	8	
EVAR	13	
Oesofagus resection	5	
Whipples operation	4	
Explorative laparotomy	3	
Retroperitoneal tumor	3	
Gastrectomy	1	
Pancreatic resection	1	
PTA + TEA	1	
Colonresection	1	

EVAR: Endovascular Aortic Repair; PTA: Percutaneous Transluminal Angioplasty; TEA: Transluminal Endarterectomy Angioplasty.

**Table 2:** Accuracy of thermometers: Comparation of performance of investigated thermometers.

Device	Mean Bias	Lower LoA	Upper LoA	Root Mean Square Deviation
Isansys lifettemp 1 min	−1.7	−3.1	−0.2	1.8
Isansys lifetemp 5 min	−1.0	−1.9	0.0	1.1
Isansys lifetemp 10 min	−0.8	−1.6	0.1	0.9
Isansys lifetemp 20 min	−0.6	−1.3	0.1	0.7
Ear thermometer Coviden genius 2	−0.1	−0.9	0.7	0.4
Temporal skin thermometer Microlife nc200	−0.1	−1.1	1.0	0.6

LoA: Limits of Agreement.

## Data Availability

Research data is available upon request.
